# Disconnection from others in autism is more than just a feeling: whole-brain neural synchrony in adults during implicit processing of emotional faces

**DOI:** 10.1186/s13229-017-0123-2

**Published:** 2017-02-22

**Authors:** Rocco Mennella, Rachel C. Leung, Margot J. Taylor, Benjamin T. Dunkley

**Affiliations:** 1 Department of Diagnostic Imaging, Hospital for Sick Children, 555 University Avenue, Toronto, Ontario M5G 1X8 Canada; 2 Department of General Psychology, University of Padova, Via Venezia 8, 35131 Padova, Italy; 3 Neurosciences & Mental Health, Hospital for Sick Children Research Institute, 555 University Avenue, Toronto, Ontario M5G 1X8 Canada; 4 Department of Medical Imaging, Faculty of Medicine, University of Toronto, 263 McCaul Street - 4th Floor, Toronto, Ontario M5T 1W7 Canada; 5 Department of Psychology, University of Toronto, 100 St. George Street, 4th Floor, Sidney Smith Hall, Toronto, Ontario M5S 3G3 Canada

**Keywords:** Autism, Functional connectivity, Magnetoencephalography, Social brain, Emotional faces, Young adults

## Abstract

**Background:**

Socio-emotional difficulties in autism spectrum disorder (ASD) are thought to reflect impaired functional connectivity within the “social brain”. Nonetheless, a whole-brain characterization of the fast responses in functional connectivity during implicit processing of emotional faces in adults with ASD is lacking.

**Methods:**

The present study used magnetoencephalography to investigate early responses in functional connectivity, as measured by interregional phase synchronization, during implicit processing of angry, neutral and happy faces. The sample (*n* = 44) consisted of 22 young adults with ASD and 22 age- and sex-matched typically developed (TD) controls.

**Results:**

Reduced phase-synchrony in the beta band around 300 ms emerged during processing of angry faces in the ASD compared to TD group, involving key areas of the social brain. In the same time window, de-synchronization in the beta band in the amygdala was reduced in the ASD group across conditions.

**Conclusions:**

This is the first demonstration of atypical global and local synchrony patterns in the social brain in adults with ASD during implicit processing of emotional faces. The present results replicate and substantially extend previous findings on adolescents, highlighting that atypical brain synchrony during processing of socio-emotional stimuli is a hallmark of clinical sequelae in autism.

**Electronic supplementary material:**

The online version of this article (doi:10.1186/s13229-017-0123-2) contains supplementary material, which is available to authorized users.

## Background

Socio-emotional deficits represent a core symptom in autism spectrum disorder (ASD) and are thought to have modulatory effects over other clinical manifestations [1]. It has been proposed that those with ASD show a lack of motivation toward social stimuli, such as emotional faces, which are considered to be naturally rewarding in typically developed (TD) individuals [1–3]. Other authors related socio-emotional difficulties in ASD to a deficit in theory of mind [4], or to a pervasive difficulty in learning stimuli’s emotional salience [5]. In the past years, different theoretical approaches have highlighted atypical activation of specific brain regions in individuals with ASD, such as the amygdalae [6, 7], insulae [8, 9], fusiform gyri [1] or superior temporal sulci [10]. Nonetheless, recently it has been suggested that ASD can be described as a neural systems disorder [11], characterized by widespread abnormalities throughout the brain [12].

Specifically, dysfunctional emotional processing and an impaired ability to recognize others’ emotions correctly have been related to disturbances in functional connectivity among brain areas important to assessing stimuli’s salience [13, 14]. In autism, this is thought to reflect atypical maturation of the “social brain”, a network of brain areas which shows co-activation across social tasks [15, 16]. The social brain includes limbic and paralimbic regions which code for different aspects of emotional significance of social stimuli, such as the amygdalae [17] and anterior hippocampi [18], the anterior insulae [19], the medial and ventromedial prefrontal cortices [16], and the anterior temporal lobes [20]. Other areas in the social brain are involved in representing shape, movement and conceptual information about animate entities (i.e. the fusiform gyri, the posterior cingulate/precuneus and regions within and near the posterior superior temporal sulci) [1, 21], in action understanding (i.e. somatosensory and anterior intraparietal cortices) [22] and social communication (i.e. the left inferior frontal gyrus) [23] (for a review see [24]). Studies using functional magnetic resonance imaging (fMRI) have reported impaired (usually decreased) connectivity in the social brain network in individuals with ASD at rest [24, 25], during processing of faces [26] and emotional faces [27, 28]. Recent evidence suggested that hypo-connectivity in autism is more evident during implicit compared to explicit emotional processing, and explicit emotional processing may be relatively preserved in high-functioning individuals with ASD due to learning and experience [28]. Since it is well known that implicit processing of emotional stimuli, such as emotional faces, relies on quick and automatized processes in the brain [29–31], a precise characterization of the time course of early brain activations in ASD is very relevant to understanding socio-emotional processing in ASD.

Compared to fMRI, magnetoencephalography (MEG) provides complementary information about the timing of the neural response, while still maintaining a good spatial resolution [32, 33]. Moreover, MEG allows the investigation of connectivity patterns at different frequency scales, which are differently modulated by emotional dimensions. In particular, emotional valence (pleasant/unpleasant) is usually represented by stimulus-related changes in higher frequencies (beta/gamma) in early time windows, whereas arousal is reflected by lower frequencies [34–42]. MEG and EEG studies consistently report beta band modulation during both explicit and implicit emotion recognition and understanding of social-emotional stimuli [43, 44]. During implicit emotional processing of dynamic facial expressions, synchronization in the beta band between fronto-limbic (i.e. pulvinar, caudate, cingulate, and amygdala) and posterior regions of the social brain (i.e. superior temporal sulcus) was observed when the facial expressions become emotionally salient [44]. This finding, along with the observed spatially concomitant fMRI BOLD response, led to the interpretation that the beta rhythm subserves feed-forward/feed-back mechanisms between fronto-limbic and posterior areas during emotional processing of socially relevant stimuli. Moreover, several studies of implicit emotional processing of faces using both intracranial recordings [45] and MEG [46, 47] suggest a role for fast gamma synchronization in subcortical limbic regions (e.g. thalamus and amygdala) in response to negatively valenced faces, followed by synchronization in both frontal and posterior areas. Accordingly, gamma synchrony has been successfully employed in assessing thalamo-cortico-limbic circuitry during implicit emotional processing of facial expressions [48].

Thus, the study of oscillatory responses in the beta and gamma ranges reliably characterizes fast spatiotemporal coupling in the social brain in response to emotional stimuli. Importantly, consistent results also come from several studies investigating implicit emotional processing of facial expressions. This is particularly relevant for the study of autism, since, as mentioned above, fMRI studies have indicated that hypo-connectivity in autism is more evident during implicit compared to explicit emotional processing [28]. Two MEG studies have examined functional connectivity during emotional face processing in ASD, and both employed an implicit emotional paradigm. Khan et al. [49] investigated MEG functional connectivity involving the fusiform gyri, in adolescents and young adults with ASD. During processing of fearful and angry faces, reduced connectivity within the right fusiform gyrus and between the fusiform and the left precuneus, left inferior frontal gyrus and left anterior cingulate was reported in ASD, within 320 ms. In accordance with these results, decreased connectivity in beta band was also seen in adolescents with ASD during implicit processing of angry faces, in the first 400 ms after the stimuli [41]. In the latter study, reduced beta connectivity in ASD was anchored in connections between visual (e.g. the fusiform) and limbic (e.g. the insula) areas involved in face-processing, consistent with the role of beta band in coupling limbic and posterior visual activation [44].

### The present study

Although there is support for early MEG under-connectivity in adolescents with ASD compared to TD, especially during processing of negatively valenced faces, a whole-brain characterization of MEG connectivity pattern in adults with ASD is lacking. Thus, in line with previous studies, we used an implicit emotional face paradigm [41] to investigate patterns of connectivity in young adults with ASD in response to angry, neutral and happy faces. Based on prior findings, we expected:

- A fast task-induced increase in connectivity in high-frequency bands (beta/gamma) in both groups [41, 42];
- Reduced connectivity within 400 ms in ASD vs. TD individuals between areas of the social brain [41, 49–51].

## Methods

### Participants

Forty-four young adults were included in the study, 22 with ASD (7 females, age = 26.4 ± 4.1) and 22 TD controls (8 females, age = 26.0 ± 3.9), matched for age and sex. All subjects had no history of neurological or neurodevelopmental disorder (other than autism), no standard contraindication for MEG and MRI, IQ ≥70, language skills adequate for task comprehension and normal or corrected to normal visual acuity. As is typical in clinical samples, the majority of ASD participants were taking standard psychotropic medications at the time of the study (e.g. Ritalin, antidepressants and anxiolytics), while controls were free from medication. All participants read and signed an informed written consent, after receiving a complete description of the study. Testing and recordings were completed in the MEG Lab at the Hospital for Sick Children, and the Research Ethics Board of the hospital gave institutional approval for the study.

### Clinical evaluation

The diagnosis of ASD was confirmed by expert clinicians, through clinical evaluation, medical reports and the Autism Diagnostic Observation Schedule-2 (ADOS-2) [52]. ASD group had a mean ADOS severity score of 6.2 ± 2.43 out of 10 [52–54]. Full-scale IQ was measured using the two-subtest form of the Wechsler Abbreviated Scale of Intelligence (WASI) [55], except for 1 ASD and 2 control participants. The two groups were matched for IQ (ASD mean = 114.0 ± 18, controls mean = 114.3 ± 10.1).

### Emotional face task in MEG

The task used in the present study has been previously described [41]. Briefly, during each trial, a face (angry, happy or neutral) was shown on either side of a central fixation cross, for 80 ms, to avoid time for any saccades; on the other side, a scrambled pattern of the face, matched for luminosity, contrast and colour, was presented simultaneously. A varying inter-trial interval (1300–1500 ms) preceded the next stimulus presentation. Participants were instructed to press left or right buttons as quickly as possible to indicate the side of the scrambled pattern, ignoring the faces. Thus, the processing of the emotional content remained implicit. The stimuli consisted of 75 colour photographs of 25 actors, posing for each of the three emotional expressions, taken from the NimStim Set of Facial expression [56]. Development of the MacBrain Face Stimulus Set was overseen by Nim Tottenham and supported by the John D. and Catherine T. MacArthur Foundation Research Network on Early Experience and Brain Development. Please contact Nim Tottenham at tott0006@tc.umn.edu for more information concerning the stimulus set. The emotional faces and their scrambled counterparts were presented in random order, twice on each side, resulting in a total of 300 trials. Response latencies were recorded. Images were back-projected onto a screen at 79 cm from the eyes, with a visual angle of 6.9°, thus in the parafoveal region.

### Neuroimaging data

MEG signal was recorded inside a magnetically shielded room, with a 151-channel CTF system (CTF-MISL, Coquitlam, BC, Canada), at a 600-Hz sampling rate. An online low-pass filter at 150 Hz was applied to the data, along with a third-order gradient cancellation. Fiducial coils on the left and right pre-auricular points and on the nasion of each subject allowed head position to be recorded continuously during the task. After the MEG, a T1-weighted MRI sequence was acquired (3D SAG MRPAGE, GRAPPA = 2, TR = 2300 ms, TE = 2.96 ms, FA = 9°, 256 × 256 matrix, 192 slices, thickness = 1 mm, isotropic voxels) with a 12-channel head coil in a 3 T MRI scanner (MAGNETOM Tim Trio, Siemens AG, Erlangen, Germany). The same fiducial points used in MEG were replaced with radio-opaque markers for the MRI, to allow for precise MEG/MRI co-registration. In both the MEG and MRI, the participants lay supine.

### Data processing and statistics

#### Behavioural data

Response latencies were entered in a mixed analysis of variance (ANOVA) with group (ASD/TD) as a between factor, and emotion (angry/happy/neutral) as a within factor. STATISTICA 7.0 software (StatSoft Inc, Tulsa, OK) was used for statistical analysis.

#### MEG data: preprocessing

MEG data were band-pass filtered offline (1–150 Hz), and a notch filter at 60 Hz and harmonics was applied. Epochs were created in the broad −1500- to 3000-ms interval around stimulus presentation, in order to prevent boundary effects, and were excluded when intra-trial head motion exceeded 5 mm. The median head position from the remaining epochs was used for source reconstruction. A single shell head model was calculated from each individual’s MRI and each individual’s brain space was normalized to a template brain (ICBM 152) [57]. The time-series of brain activation for the 90 seeds of the Automated Anatomical Labeling (AAL) atlas [58] were estimated through a linearly constrained minimum variance (LCMV) beamformer [59]. Beamformers act as spatial filters, which reconstruct the signal from the desired region of interest (ROI) while suppressing signals from all other unwanted sources [60]. Due to the minimization of the contribution of the sources outside the ROI, beamformers are effective at suppressing ocular and non-ocular artefacts, therefore not requiring trial-by-trial artefact rejection based on visual inspection [61]. Moreover, as detailed below, the connectivity measure which was employed in the present study (the phase lag index; PLI) protects from potentially residual noise from common sources.

#### MEG data: functional connectivity

The time-series from each ROI were filtered into canonical bands (theta 4–7 Hz; alpha 8–14; beta 15–30; gamma 30–80), and a Hilbert transform was applied to the filtered data to extract the instantaneous phase at each time sample for each frequency. The PLI was calculated to estimate the cross-trial degree of phase synchronization between all pairwise combinations of the seeds for every time point, resulting in a 90 × 90 adjacency matrix for each subject, at each sample. The PLI quantifies the phase synchrony by calculating the asymmetry of the distribution of the instantaneous phase differences between two time series. It ranges from 0 (completely symmetric, or random, phase distribution) to 1 (completely asymmetric phase distribution); since field/spread volume conduction or leakage of common sources would determine a zero (or 180°) phase lag between the two sources, this measure is unaffected by spurious coupling that could be generated by artefacts instantaneously projecting to each of the ROIs.

**Fig. 1:**
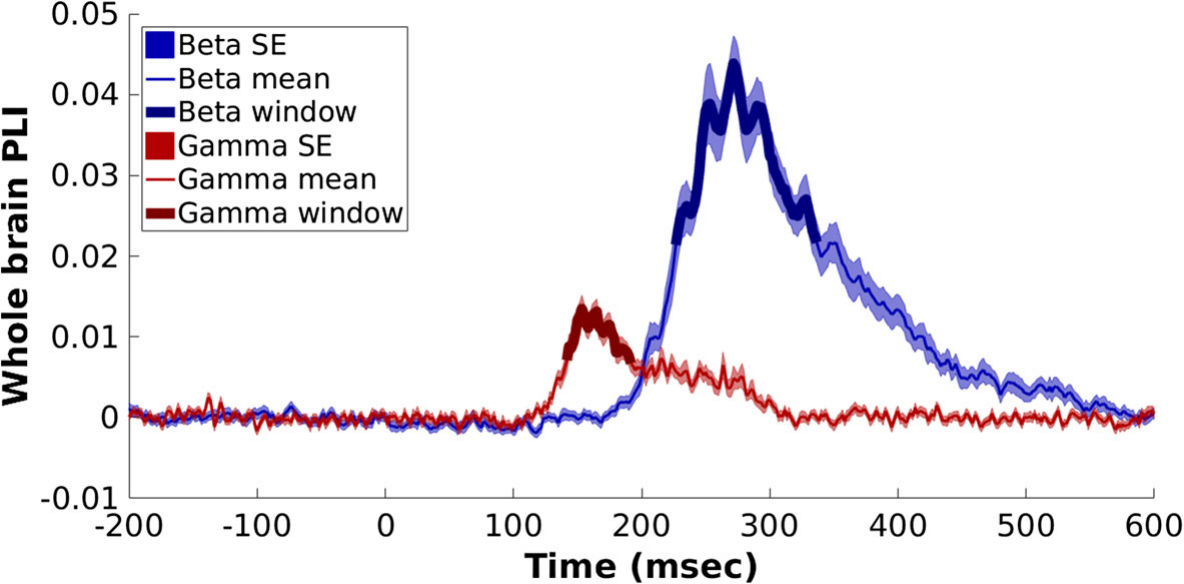
Mean “whole brain” connectivity response in beta and gamma bands across groups and conditions. The time windows selected for further analyses are represented with *thicker lines*

PLI values from each participant’s 90 × 90 matrix at each time point were averaged, to extract the time course of the mean “whole-brain” (entire seed grid) response in functional connectivity. The values were baselined (−200–0 ms), and a grand average across groups and condition was computed. Beta and gamma connectivity peaked within 300 ms, as expected considering their role in rapid brain response to emotional faces [42]. To determine the specificity of this effect, peaks in alpha and theta band connectivity were also computed and both resulted in peak connectivity after 400 ms, thus, after the time of interest for the present study. Therefore, as the aim of the study was investigating functional connectivity responses during quick and automatized implicit processing of emotional stimuli (<400 ms), we selected beta and gamma bands for further analyses. To determine the time windows of interest for the statistical analyses, a data-driven approach was chosen. In order to focus on the main response, the time window of interest was defined as the full duration at half maximum—the temporal equivalent of FWHM—centred on the peak in PLI. Thus, for beta band we focused on the 227–337-ms interval and for gamma band on the 142–192-ms interval (Fig. 1). Based on these time windows, a temporally averaged adjacency matrix was generated separately for each frequency and emotion. The network-based statistics (NBS) [62] was used to compute statistical contrasts within groups (active window vs. baseline) and between groups (active windows, ASD vs. TD). NBS considers the connectivity matrix in terms of graph theory, accounting for their topological distribution: first, a test statistic for each individual connection is computed (a *t* test in the present study); the matrix is then thresholded and any connected structures (i.e. *components*) that might be present in the supra-threshold connections are identified. Thus, a component is defined as contiguous groups of network nodes bound by suprathreshold connections. A *p* value corrected by Family Wise Error rate is assigned to each component, by means of permutation testing, as follows. For each permutation (*n* = 5000 in the present study), the group/condition to which each participant belongs is shuffled randomly and the corresponding maximal component size is calculated and stored. Thus, an empirical estimate of the null distribution of the maximal component size is generated. Finally, the number of permutations for which the maximal component size is greater than the original one, normalized for the total number of permutations, determines the *p* value of the component.

The threshold for the *t*-statistic for the initial univariate testing was adapted to the data, as suggested by Zalesky and colleagues [62]. Therefore, for within contrasts, thresholding values were set to 7.5 and 5 for beta and gamma bands, respectively. For between contrasts, a value of 3.1 was used for both bands. The importance of each node was assessed using the node strength measure (i.e. the sum of weights of links connected to the node), computed using the Brain Connectivity Toolbox [63]. Results were considered to be statistically significant at *p* < 0.05, corrected. Both the significant components and the node strength data were visualized using BrainNet Viewer [64].

#### MEG data: time-frequency analyses

To complement the whole-brain connectivity findings, event-related synchronization/de-synchronization (ERS/D) were explored in specific seeds of interest: seeds of interest were selected among network nodes involved in the significant component that emerged from between-groups statistical contrasts with NBS. In particular, seed selection was based on the literature of brain regions involved in processing of emotional faces in both TD and ASD individuals. A Morlet wavelet transformation, as implemented in Fieldtrip [65], on each selected seed’s estimated time-series was applied; the proportion of change relative to the baseline (−200 to 0 ms) in each frequency bin at each 10-ms time point was computed.

## Results

### Response latency

Neither the main effects for group and emotion, nor the group × emotion interaction were significant for the response latency (all *p* > 0.21), indicating that both groups performed equally well on the task.

### Connectivity: within groups

#### Beta band

**Fig. 2:**
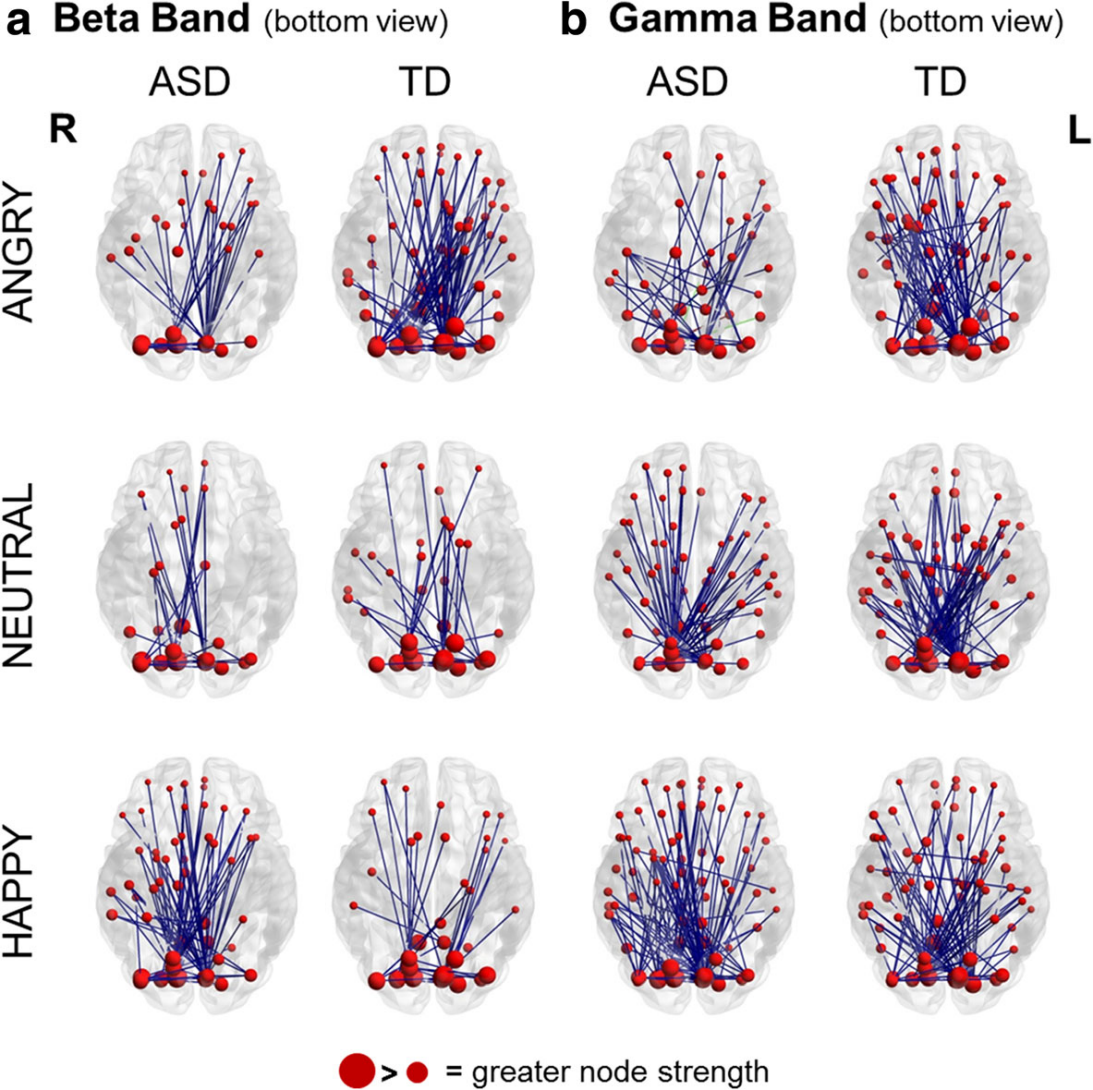
Task-dependent increases in connectivity for each group and condition. **a** Task-dependent increases in beta band; **b** task-dependent increases in gamma band. *Blue lines* represent significant connections between seeds. The brains are viewed from the bottom. R = right, L = left, ASD = autism group, TD = typically developed group

Both controls and ASD participants displayed a significant increase in beta connectivity in the active window compared to baseline, for each emotion condition (Fig. 2, *left*; Additional file 1: Table S1). Across groups and conditions, occipital areas involved in basic visual processing (e.g. calcarine sulcus, cuneus) were among the most connected seeds (Additional file 2: Table S2). Both groups showed also an involvement of areas related to the core face processing system [50, 66], such as the inferior occipital and the lingual gyri. The increased connectivity also included regions of the emotional system for face processing (amygdala, insula, and striatum (i.e. pallidum, caudate and putamen in the AAL)). The face-related extended system in the model from Gobbini and Haxby includes both the emotional system and other brain areas which participate in retrieval of different aspects of person knowledge (i.e. anterior and posterior cingulate, precuneus, posterior superior temporal sulcus/temporo-parietal junction, and anterior temporal cortices) [50], which were also involved in increased beta connectivity.

#### Gamma band

Control and ASD participants also displayed a significant increase in gamma connectivity in the active window compared to baseline, for each condition (Fig. 2, *right*; Additional file 1: Table S1). For the gamma band, the calcarine sulcus and the cuneus were highly connected, as well as the nodes of the core, extended and, in particular, emotion systems for face processing. Of note, in contrast to the beta band results, the fusiform gyri, and in particular the right fusiform, were almost always present among the nodes involved in increased gamma connectivity across groups and conditions (Additional file 3: Table S3).

### Connectivity: between groups

#### Beta band

**Fig. 3:**
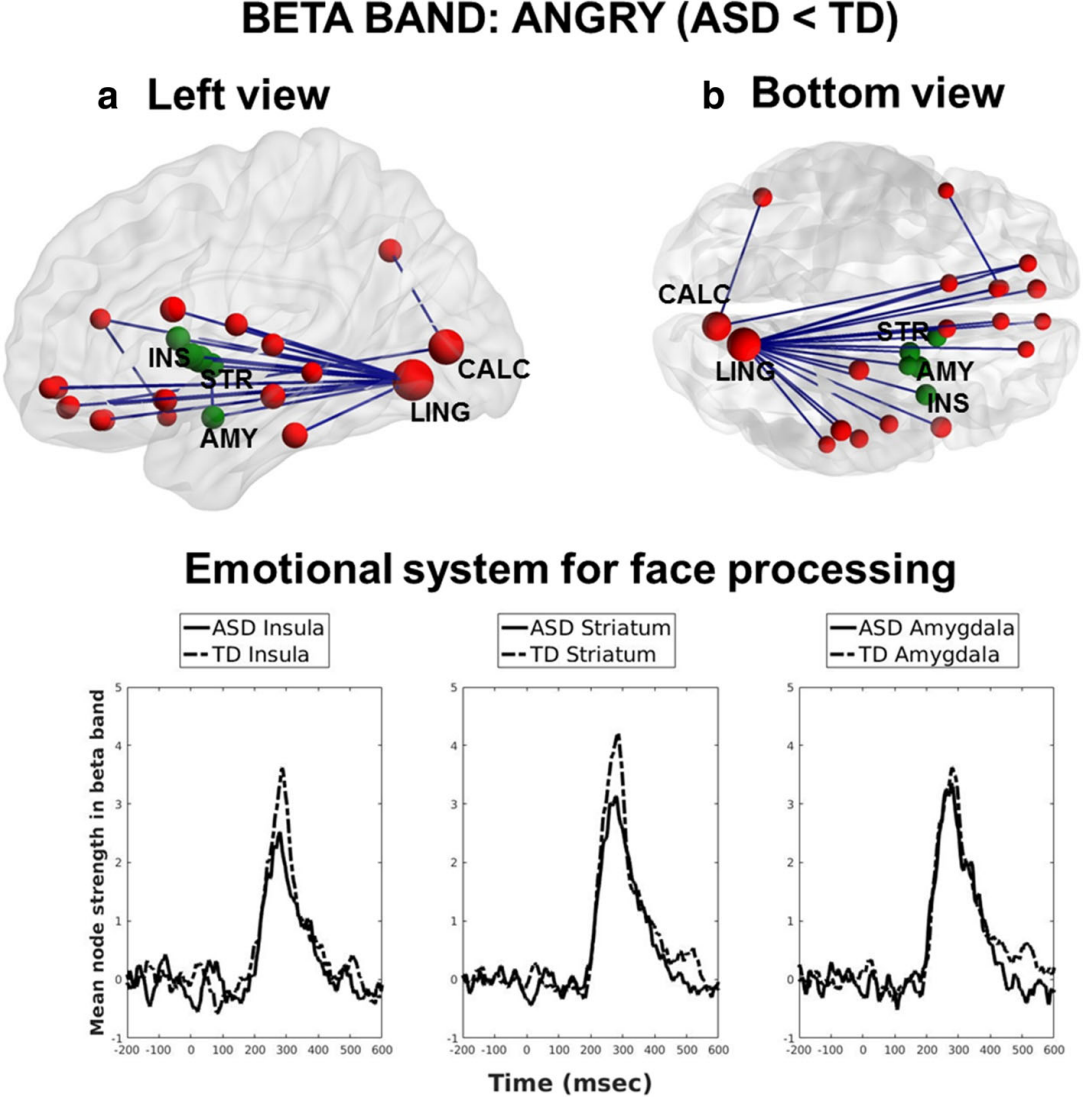
Between-group comparison of phase synchronization in the beta band, during processing of angry faces. **a** Left and **b** bottom view of reduced beta band interregional phase-locking in ASD individuals compared to TD controls. In green, seeds from the emotional system for face processing. *Bottom* time course of node strength of the seeds within the emotional system (smoothed for clarity with a 30-ms moving average)

From the contrasts between groups, a difference emerged for the processing of angry faces. Specifically, participants with ASD showed reduced beta interregional phase-locking compared to controls (*p* = 0.04, corrected). Reduced beta connectivity involved mainly connections of frontal and limbic areas with the calcarine sulcus and the lingual gyrus in the occipital lobe (Fig. 3, *top*; Table 1). The reduction in connectivity in ASD emerged for all the areas of the emotion system for face processing, within the left hemisphere (Fig. 3, *bottom*). Overall, the network nodes involved were largely left-lateralized, apart from a few frontal and parietal sites. No significant results emerged for the between-group contrasts in the happy or neutral condition.

**Table 1:** Beta hypo-connectivity in ASD compared to TD individuals: involved areas, number of connected seeds and group difference in node strength (TD-ASD)

Area	Hemisphere (L/R)	Number of connections	∆ Node strength (TD-ASD)
Lingual	L	20	2.74
Calcarine	L	2	1.89
Pallidum	L	2	0.50
Amygdala	L	2	0.22
Superior orbitofrontal cortex	R	2	0.02
Anterior cingulate cortex	R	2	−0.11
Putamen	L	1	0.72
Insula	L	1	0.61
Frontal inferior operulum	L	1	0.57
Inferior temporal gyrus	L	1	0.55
Hippocampus	L	1	0.47
Caudate	L	1	0.39
Angular gyrus	L	1	0.26
Superior temporal gyrus	L	1	0.16
Rolandic operculum	L	1	0.16
Olfactory cortex	L	1	0.11
Gyrus rectus	R	1	0.00
Olfactory cortex	R	1	−0.02
Gyrus rectus	L	1	−0.07
Middle temporal gyrus	L	1	−0.08
Superior orbitofrontal cortex	L	1	−0.14
Medial orbitofrontal cortex	L	1	−0.19
Superior temporal pole	R	1	−0.27
Medial orbitofrontal cortex	R	1	−0.33

#### Gamma band

None of the contrasts reached statistical significance.

### Time-frequency responses

Since a pattern of reduced connectivity emerged in the ASD group in the beta band, we investigated the time-frequency responses in the beta band in specific seeds of interest, as defined in the “Methods” section. Based on the large literature on TD and ASD individuals and the importance of amygdalae and insulae in processing of emotional faces [51], we focused on these seeds. Moreover, the majority of the connections involved the lingual gyrus, which, due to its importance in face processing [66], was also included for further investigation. All the selected sites were left-lateralized, in line with the between-group connectivity results.

**Fig. 4:**
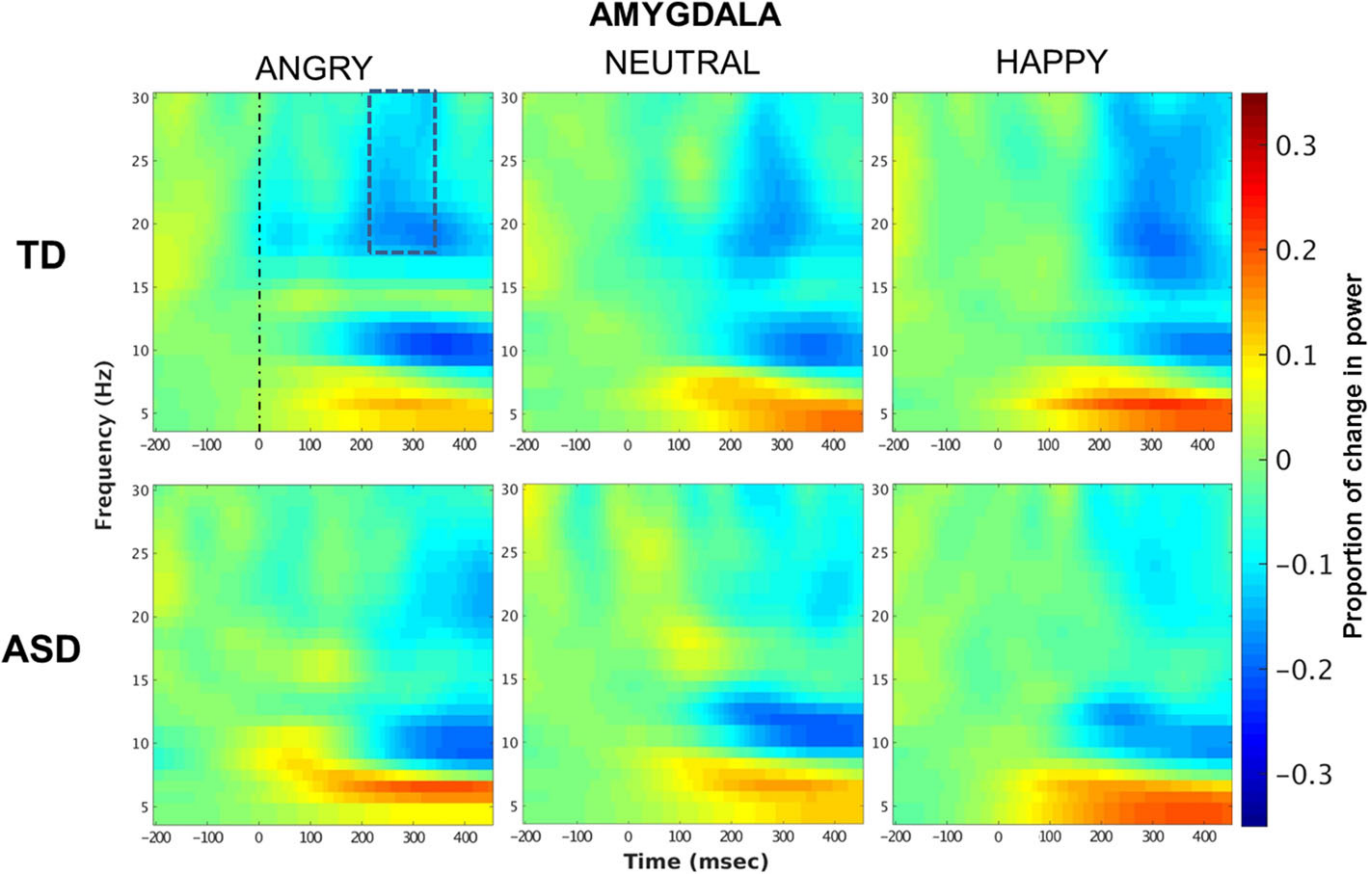
Left amygdala: time-frequency activation. Beta de-synchronization occurred in a time window overlapping with the connectivity peak (*blue scattered line*). Beta ERD in the amygdala was smaller (less negative) in ASD compared to TD across stimuli (*p* = 0.003)

Across seeds there was a general pattern of de-synchronization in the beta band overlapping in time with the peak in the connectivity response (Fig. 4; Additional file 4: Figure S1). Thus, we averaged beta ERD values in time, between 220 and 340 ms, and in frequency, between 18 and 30 Hz, on each selected seed. Mean values were entered in a mixed ANOVA with group as a between-group factor, and emotion as a within-group factor. A significant effect for group emerged in the amygdala (*F*_(1,42)_ = 9.68, *p* = 0.003) indicating that ERD was reduced in ASD compared to TD irrespective of stimuli’s category. No other main effects/interactions emerged for amygdala, insula nor lingual seeds (all *p* > 0.09).

## Discussion

To the best of our knowledge, this is the first study investigating whole-brain MEG connectivity in adults with ASD compared to TD controls during implicit processing of emotional faces. The ASD group showed reduced connectivity in the beta band compared to TD subjects during implicit processing of angry faces. In particular, reduced connectivity in ASD involved several areas of the social brain. Specifically, both low-level visual areas, such as the calcarine cortex, and face-sensitive regions, such as the lingual gyrus and the inferior temporal gyrus [66], were significantly hypo-connected in ASD compared to TD individuals. Other regions pertaining to the extended system for face processing [50], such as the superior temporal gyrus, the temporal pole and the anterior cingulate cortex, were also under-connected in ASD. Importantly, the network included the amygdala, the insula and the striatum, which are central in the emotional processing of faces. Of note, these structures are deep and/or subcortical regions of the brain; therefore, the present results align with the large body of MEG literature which supports the use of beamforming algorithms for MEG source reconstruction of subcortical neural activity [41, 46, 67–75]. Based on beamforming solutions, functional connectivity within thalamo-cortico-limbic circuits has been studied using MEG during processing of emotional faces [48]. Moreover, our results largely converge with recent fMRI findings which reported reduced connectivity between limbic (e.g. amygdala) and cortical regions of the social brain in ASD compared to TD individuals, during both explicit [27] and implicit [28] processing of emotional faces. The present study adds to the literature by providing a precise characterization in the time domain of reduced functional connectivity in ASD individuals.

The majority of the seeds involved were lateralized to the left hemisphere. A model has been proposed that anterior regions of the left cerebral hemisphere subtend *approach* motivation, while the anterior regions of the right hemisphere are responsible for *withdrawal* motivation [76–82]. It has been shown that, although a negatively valenced emotion, anger elicits approach motivation and behaviours, associated with greater left than right brain activation [83–87]. Thus, hypo-connectivity in the left hemisphere during processing of angry faces supports the theory of poor motivation toward social stimuli in ASD compared to TD individuals [1–3]. Intriguingly, our findings largely overlap with results from Leung and colleagues who used the same task on adolescents with autism (range 12–15 years) [41]. Nonetheless, the latter study did not find the strong left lateralization for angry faces. This suggests that hemispheric specialization in emotional face processing evolves during the development and ASD individuals may show an atypical developmental trajectory. Supporting this interpretation, subjects with ASD show an opposite pattern of MEG lateralization compared to TD individuals for other cognitive functions, such as language. While TD individuals showed a shift toward left-lateralization for language as age increased, those with ASD showed the opposite pattern, that is a right-sided lateralization with age [88]. This is in line with structural studies which highlight a shift toward right lateralization in cortical volume in ASD [89]. Interpretations of laterality effects, however, ought to be taken with caution, since no statistical tests on connectivity strength at left and right sites were run. Beamformers are not ideal for estimation of stimulus-driven oscillatory activity of bilateral sources, which can be highly synchronized [90], and therefore for testing laterality effects. The supposition that differences exist in the developmental trajectory of emotional processing should be investigated using other source localization algorithms as well, and by research comparing connectivity asymmetries at different ages, or longitudinally.

Consistent with other research, under-connectivity in social brain areas in ASD emerged for angry, but not happy faces [41, 49]. Behavioural studies have shown that autism is associated with specific difficulties in processing of negative emotional faces [91], and in particular angry faces [92]. In a previous study on adolescents with ASD [41], hypo-connectivity for angry faces had been related to the observation that the ability to identify negative emotions, such as anger and fear, matures at later ages, and therefore negative emotions may require more complex processing compared to positive emotions [93–96]. This development-related explanation may not hold for our results with adults with ASD. Another possible interpretation relates to angry faces being negatively valenced social stimuli, which convey information about potential danger or harm for the individual. Thus, compared to positively valenced stimuli, they are thought to be processed very quickly in the brain, allowing the organism to prepare a rapid behavioural response [42, 78, 97]. Therefore, it is plausible that atypical functional connectivity in ASD emerged due to deficits in the high-demanding quick processing requirements of negative content, especially in the context of our very rapidly presented implicit processing task.

The present results also extend previous EEG/MEG studies showing the importance of beta band in processing of emotional valence of social and non-social emotional stimuli [35, 42, 98]. In particular, we replicated the increase in signal coherence within the beta band reported during processing of visual emotional stimuli [39, 41]. Moreover, by combining the connectivity analysis with a time-frequency analysis on seeds of interests, it emerged that the increase in beta connectivity overlapped in time with a decrease in beta power in the 220–340-ms window. Our results align with studies showing that event-related beta power correlates negatively with the hemodynamic response in task-relevant brain areas [99, 100]. Beta de-synchronization has been reported during processing of salient external events [101], both in EEG [43, 102] and MEG studies [103], and it has been interpreted as an adaptation process in response to the perceived emotional information [43]. In the present study, beta ERD in the left amygdala was reduced in ASD compared to TD individuals irrespective of stimulus condition, suggesting that participants with ASD had a less pronounced response to stimulus salience. Overall, we propose that the general difficulty in extracting emotional information from faces in ASD, as indicated by amygdala hypo-responsivity, is associated with impaired long-range communication between areas of the social brain, which instead is specific for highly salient angry faces.

It was surprising that the involvement of the fusiform gyri did not emerge in the connectivity differences between ASD and TD individuals. This may be attributed to the timing of the beta peak in connectivity, which occurred after the time window where the fusiform activation is usually maximal (i.e. 130–200 ms) [104]. As a confirmation of this hypothesis, the within group results showed that the fusiform was more involved in the gamma response to the task, which peaked between 142 and 192 ms. Previous research demonstrated that, during processing of human faces, gamma band is associated with “structural encoding”, which represents the integration of the configural metrics and face-feature characteristics into a detailed representation [105]. Other MEG studies also found that face-induced gamma oscillations, albeit not representing the same neurophysiological process as the M170, were generated by common sources, including the right fusiform gyrus [106]. Although connectivity in gamma band in autism during processing of emotional faces has been rarely investigated, one study reported differences in gamma power during emotional face processing in ASD compared to TD individuals [103]. Thus, while the group effect in the gamma band connectivity was not significant in the present study, future studies should further clarify the role of gamma band in emotional face processing in ASD.

## Conclusions

The present study provides the first demonstration of hypo-connectivity in adults with ASD compared to TD individuals during emotional face processing, using a whole-brain MEG approach. Under-connectivity in ASD was evident in the beta band during processing of angry faces and involved key areas of the social brain largely in the left hemisphere, such as the amygdala, the insula and the lingual gyrus. Complementary time-frequency analyses highlighted hypo-activation of the amygdala which, together with the pattern of under-connectivity in the social brain, could contribute to the socio-emotional difficulties in ASD. These findings shed light on important neural underpinnings of the disorder, providing potential for the development of new techniques to aid in the diagnosis and assessment of the efficacy of treatments.

## Additional files

Additional file 1: Table S1. Significant NBS components for beta and gamma bands in ASD and TD group (active window vs. baseline). (DOCX 15 kb)
Additional file 2: Table S2. Within connectivity in the beta band: Number of connections for each AAL node within the significant NBS components. Nodes are ordered by the sum of the connections across groups and conditions (from the greater to the smaller value). (DOCX 18 kb)
Additional file 3: Table S3. Within connectivity in the gamma band: Number of connections for each AAL node within the significant NBS components. Nodes are ordered by the sum of the connections across groups and conditions (from the greater to the smaller value). (DOCX 22 kb)
Additional file 4: Figure S1. Time-frequency activation: Beta de-synchronization in the left lingual gyrus and insula. (TIF 1151 kb)

## Abbreviations

AAL: Automated Anatomical Labeling
ADOS-2: Autism Diagnostic Observation Schedule-2
ANOVA: Analysis of variance
ASD: Autism spectrum disorder
EEG: Electroencephalography
ERS/D: Event-related synchronization/de-synchronization
fMRI: Functional magnetic resonance imaging
FWHM: Full-width at half maximum
IQ: Intelligence quotient
LCMV: Linearly constrained minimum variance
MEG: Magnetoencephalography
MRI: Magnetic resonance
NBS: Network-based statistics
PLI: Phase lag index
ROI: Region of interest
TD: Typically developed
WASI: Wechsler Abbreviated Scale of Intelligence
