# Supplementary material for: Disconnection from others in autism is more than just a feeling: whole-brain neural synchrony in adults during implicit processing of emotional faces
Source: Mol Autism. 2017 Feb 22;8:7. doi: 10.1186/s13229-017-0123-2 (PMC5351200; doi:10.1186/s13229-017-0123-2)
Supplement: Additional file 1: Table S1. — Significant NBS components for beta and gamma bands in ASD and TD group (active window vs. baseline). (DOCX 15 kb) [file 13229_2017_123_MOESM1_ESM.docx]

**Table S1.** Significant NBS components for beta and gamma bands in ASD and TD group (active window vs. baseline).

| **BAND** | **GROUP** | **Condition** | **N components** | **N nodes** | **N edges** |
| --- | --- | --- | --- | --- | --- |
| BETA | TD | Angry | 1 | 58 | 94 |
|  |  | Neutral | 1 | 31 | 30 |
|  |  | Happy | 1 | 32 | 32 |
|  | ASD | Angry | 2 | 30 | 33 |
|  |  | Neutral | 1 | 24 | 30 |
|  |  | Happy | 3 | 49 | 72 |
| GAMMA | TD | Angry | 1 | 56 | 83 |
|  |  | Neutral | 1 | 55 | 86 |
|  |  | Happy | 1 | 49 | 59 |
|  | ASD | Angry | 3 | 37 | 45 |
|  |  | Neutral | 1 | 56 | 68 |
|  |  | Happy | 1 | 70 | 109 |
