# Supplementary material for: Disconnection from others in autism is more than just a feeling: whole-brain neural synchrony in adults during implicit processing of emotional faces
Source: Mol Autism. 2017 Feb 22;8:7. doi: 10.1186/s13229-017-0123-2 (PMC5351200; doi:10.1186/s13229-017-0123-2)
Supplement: Additional file 2: Table S2. — Within connectivity in the beta band: Number of connections for each AAL node within the significant NBS components. Nodes are ordered by the sum of the connections across groups and conditions (from the greater to the smaller value). (DOCX 18 kb) [file 13229_2017_123_MOESM2_ESM.docx]

Table S2. Within connectivity in the beta band: Number of connections for each AAL node within the significant NBS components. Nodes are ordered by the sum of the connections across groups and conditions (from the greater to the smaller value).

|  |  | **BETA BAND** | | | | | |
| --- | --- | --- | --- | --- | --- | --- | --- |
|  |  | **TD** | | | **ASD** | | |
| **AAL Seed** | **L/R** | **ANGRY** | **NEUTRAL** | **HAPPY** | **ANGRY** | **NEUTRAL** | **HAPPY** |
| Cuneus | L | 11 | 6 | 5 | 18 | 13 | 20 |
| Calcarine Sulcus | L | 22 | 4 | 4 | 3 | 1 | 11 |
| Cuneus | R | 11 | 2 | 2 | 4 | 5 | 20 |
| Calcarine Sulcus | R | 7 | 3 | 7 | 4 | 3 | 7 |
| Superior Occipital Gyrus | R | 17 | 2 | 4 | 2 | 3 | 2 |
| Inferior Occipital Gyrus | R | 11 | 1 | 4 | 3 | 4 | 5 |
| Middle Occipital Gyrus | L | 6 | 6 | 2 |  | 2 |  |
| Superior Occipital Gyrus | L | 5 | 2 | 3 | 3 | 1 | 2 |
| Lingual Gyrus | L | 7 | 2 | 3 |  | 1 | 1 |
| Olfactory Gyrus | L | 4 | 4 | 1 | 2 |  | 2 |
| Precuneus | R | 2 |  | 2 |  | 5 | 3 |
| Inferior Occipital Gyrus | L | 2 | 1 | 2 | 1 | 1 | 4 |
| Precuneus | L | 6 | 1 | 3 |  |  | 1 |
| Amygdala | L | 4 | 1 |  | 1 |  | 2 |
| Caudate Nucleus | L | 3 | 2 |  | 1 |  | 2 |
| Inferior Frontal Gyrus, pars orbitalis | L | 5 |  | 1 | 2 |  |  |
| Lingual Gyrus | R |  | 6 | 1 |  |  | 1 |
| Medial Orbitofrontal Cortex | R | 2 | 1 | 2 |  |  | 3 |
| Middle Occipital Gyrus | R | 2 |  |  | 2 | 1 | 3 |
| Rectus Gyrus | L | 2 |  | 1 |  | 3 | 2 |
| Hippocampus | R |  | 1 |  |  | 4 | 2 |
| Pallidum | L | 4 | 2 |  | 1 |  |  |
| ParaHippocampal Gyrus | L | 5 |  | 1 | 1 |  |  |
| Middle Frontal Gyrus, orbital part | L | 3 | 1 |  | 1 |  | 1 |
| Middle Temporal Pole | L |  |  | 2 | 2 |  | 2 |
| Olfactory Gyrus | R | 1 |  | 1 | 1 | 1 | 2 |
| Heschl Gyrus | R |  |  |  | 1 |  | 4 |
| Superior Frontal Gyrus, orbital part | L | 3 |  |  | 2 |  |  |
| Superior Temporal Pole | L | 1 |  |  | 2 |  | 2 |
| Angular Gyrus | R | 3 |  |  |  | 1 |  |
| Inferior Frontal Gyrus, pars opercularis | L | 2 |  | 2 |  |  |  |
| Insula | L | 3 |  | 1 |  |  |  |
| Medial Orbitofrontal Cortex | L | 1 | 1 |  |  | 2 |  |
| Midcingulate Gyrus | R |  | 1 |  |  |  | 3 |
| Middle Frontal Gyrus | L |  |  |  |  |  | 4 |
| Middle Frontal Gyrus | R | 1 |  |  |  | 2 | 1 |
| Middle Frontal Gyrus, orbital part | R | 2 | 1 |  |  |  | 1 |
| Precentral Gyrus | R | 1 | 1 | 1 | 1 |  |  |
| Rectus Gyrus | R | 1 |  |  | 1 |  | 2 |
| Superior Frontal Gyrus, orbital part | R | 1 |  | 1 |  | 1 | 1 |
| Supplementary Motor Area | R |  | 1 |  | 1 |  | 2 |
| Caudate Nucleus | R |  |  | 1 |  | 1 | 1 |
| Inferior Frontal Gyrus, pars triangularis | L | 2 |  | 1 |  |  |  |
| Midcingulate Gyrus | L | 1 |  |  |  | 2 |  |
| Middle Temporal Gyrus | R | 1 |  |  |  |  | 2 |
| Pallidum | R |  |  |  | 1 |  | 2 |
| Postcentral Gyrus | R | 1 |  |  |  |  | 2 |
| Posterior Cingulate Gyrus | L | 1 |  | 1 |  |  | 1 |
| Putamen | R |  |  |  | 1 |  | 2 |
| Superior Parietal Gyrus | L |  |  | 1 |  |  | 2 |
| Superior Temporal Gyrus | R |  |  |  | 1 |  | 2 |
| Thalamus | R | 1 |  |  | 1 |  | 1 |
| Amygdala | R | 1 |  |  |  |  | 1 |
| Angular Gyrus | L | 1 | 1 |  |  |  |  |
| Anterior Cingulate Gyrus | L |  | 1 |  | 1 |  |  |
| Anterior Cingulate Gyrus | R | 1 |  |  |  | 1 |  |
| Fusiform Gyrus | L | 1 |  |  |  |  | 1 |
| Inferior Parietal Gyrus | R | 1 | 1 |  |  |  |  |
| Insula | R |  |  |  |  |  | 2 |
| Medial Frontal Gyrus | L | 1 |  |  |  |  | 1 |
| Medial Frontal Gyrus | R | 1 |  |  |  |  | 1 |
| Paracentral Lobule | L | 1 | 1 |  |  |  |  |
| Paracentral Lobule | R | 1 | 1 |  |  |  |  |
| ParaHippocampal Gyrus | R |  |  |  |  | 1 | 1 |
| Putamen | L | 2 |  |  |  |  |  |
| Rolandic Operculum | L | 2 |  |  |  |  |  |
| Superior Frontal Gyrus | L |  |  |  |  |  | 2 |
| Superior Parietal Gyrus | R | 1 |  |  |  | 1 |  |
| Supramarginal Gyrus | R | 1 | 1 |  |  |  |  |
| Fusiform Gyrus | R |  |  |  |  |  | 1 |
| Heschl Gyrus | L |  |  |  | 1 |  |  |
| Hippocampus | L |  |  | 1 |  |  |  |
| Inferior Frontal Gyrus, pars orbitalis | R |  |  | 1 |  |  |  |
| Inferior Frontal Gyrus, pars triangularis | R |  |  |  |  |  | 1 |
| Inferior Parietal Gyrus | L | 1 |  |  |  |  |  |
| Inferior Temporal Gyrus | R |  |  | 1 |  |  |  |
| Middle Temporal Gyrus | L |  |  | 1 |  |  |  |
| Posterior Cingulate Gyrus | R | 1 |  |  |  |  |  |
| Precentral Gyrus | L | 1 |  |  |  |  |  |
| Rolandic Operculum | R |  | 1 |  |  |  |  |
| Superior Temporal Gyrus | L | 1 |  |  |  |  |  |
| Thalamus | L | 1 |  |  |  |  |  |
| Inferior Frontal Gyrus, pars opercularis | R |  |  |  |  |  |  |
| Inferior Temporal Gyrus | L |  |  |  |  |  |  |
| Middle Temporal Pole | R |  |  |  |  |  |  |
| Postcentral Gyrus | L |  |  |  |  |  |  |
| Superior Frontal Gyrus | R |  |  |  |  |  |  |
| Superior Temporal Pole | R |  |  |  |  |  |  |
| Supplementary Motor Area | L |  |  |  |  |  |  |
| Supramarginal Gyrus | L |  |  |  |  |  |  |
