# Supplementary material for: Disconnection from others in autism is more than just a feeling: whole-brain neural synchrony in adults during implicit processing of emotional faces
Source: Mol Autism. 2017 Feb 22;8:7. doi: 10.1186/s13229-017-0123-2 (PMC5351200; doi:10.1186/s13229-017-0123-2)
Supplement: Additional file 3: Table S3. — Within connectivity in the gamma band: Number of connections for each AAL node within the significant NBS components. Nodes are ordered by the sum of the connections across groups and conditions (from the greater to the smaller value). (DOCX 22 kb) [file 13229_2017_123_MOESM3_ESM.docx]

**Table S3. Within connectivity in the gamma band:** Number of connections for each AAL node within the significant NBS components. Nodes are ordered by the sum of the connections across groups and conditions (from the greater to the smaller value).

|  | | **GAMMA BAND** | | | | | |
| --- | --- | --- | --- | --- | --- | --- | --- |
|  |  | **TD** | | | **ASD** | | |
| **AAL seeds** | **L/R** | **ANGRY** | **NEUTRAL** | **HAPPY** | **ANGRY** | **NEUTRAL** | **HAPPY** |
| Cuneus | R | 9 | 25 | 13 | 2 | 32 | 20 |
| Cuneus | L | 12 | 10 | 3 | 11 | 1 | 37 |
| Calcarine Sulcus | L | 17 | 21 | 7 | 4 | 12 | 7 |
| Calcarine Sulcus | R |  | 3 | 10 | 7 | 15 | 6 |
| Superior Occipital Gyrus | R | 4 | 14 | 4 | 1 | 2 | 15 |
| Superior Occipital Gyrus | L | 7 | 3 | 13 | 3 | 1 | 9 |
| Middle Occipital Gyrus | R | 5 | 4 | 2 | 4 | 2 | 10 |
| Lingual Gyrus | L | 11 |  | 2 |  | 3 | 4 |
| Lingual Gyrus | R | 6 | 4 | 2 | 2 | 6 |  |
| Middle Occipital Gyrus | L | 4 | 2 | 2 | 3 | 1 | 5 |
| Inferior Occipital Gyrus | L | 2 | 4 | 2 | 2 |  | 6 |
| Precuneus | R | 3 | 3 | 1 | 2 | 4 | 2 |
| Anterior Cingulate Gyrus | R | 2 | 2 | 2 | 5 | 1 | 2 |
| Putamen | R | 5 | 2 | 1 |  |  | 6 |
| Pallidum | R | 5 | 2 |  |  | 1 | 4 |
| Hippocampus | L | 1 | 4 | 2 | 1 | 2 | 1 |
| Inferior Occipital Gyrus | R | 3 |  | 5 |  | 1 | 2 |
| Angular Gyrus | R | 1 | 1 | 1 | 4 | 1 | 2 |
| Precentral Gyrus | R | 5 | 3 | 1 |  |  | 1 |
| Caudate Nucleus | R | 4 |  | 3 |  | 1 | 1 |
| Putamen | L | 1 | 4 | 2 | 1 | 1 |  |
| Amygdala | L | 1 | 3 | 3 |  | 1 |  |
| Heschl Gyrus | R |  | 1 |  | 6 | 1 |  |
| Medial Frontal Gyrus | R | 2 | 3 |  |  |  | 3 |
| Middle Temporal Gyrus | R |  | 2 |  | 2 |  | 4 |
| Postcentral Gyrus | R | 3 | 1 | 2 |  |  | 2 |
| Thalamus | R | 2 | 1 |  | 2 | 1 | 2 |
| Angular Gyrus | L | 3 |  | 2 | 1 | 1 |  |
| Fusiform Gyrus | L | 1 | 1 | 2 | 1 | 2 |  |
| Fusiform Gyrus | R | 2 | 2 | 2 |  | 1 |  |
| Insula | L |  | 2 | 1 | 1 | 2 | 1 |
| Insula | R | 1 | 4 |  |  |  | 2 |
| Midcingulate Gyrus | L | 2 | 1 | 1 |  |  | 3 |
| Precuneus | L |  |  |  | 4 | 1 | 2 |
| Thalamus | L | 2 | 2 |  | 1 | 2 |  |
| Anterior Cingulate Gyrus | L | 1 | 1 | 1 | 1 | 1 | 1 |
| Hippocampus | R |  | 3 | 1 |  | 2 |  |
| Inferior Frontal Gyrus, pars opercularis | L |  | 1 |  | 3 | 1 | 1 |
| Inferior Temporal Gyrus | R |  | 2 |  | 1 |  | 3 |
| Midcingulate Gyrus | R |  | 3 |  |  | 1 | 2 |
| Paracentral Lobule | L | 1 | 1 | 1 | 1 | 1 | 1 |
| Postcentral Gyrus | L |  | 1 |  | 2 | 2 | 1 |
| Posterior Cingulate Gyrus | R | 2 |  | 2 |  |  | 2 |
| Superior Temporal Pole | R | 3 |  |  |  | 2 | 1 |
| Supplementary Motor Area | R |  | 3 |  |  |  | 3 |
| Inferior Frontal Gyrus, pars orbitalis | R | 4 |  |  |  | 1 |  |
| Inferior Frontal Gyrus, pars triangularis | L |  |  |  | 1 | 2 | 2 |
| Inferior Parietal Gyrus | L |  |  | 1 | 1 | 1 | 2 |
| Middle Frontal Gyrus, orbital part | L | 2 |  | 2 |  |  | 1 |
| Middle Frontal Gyrus, orbital part | R | 1 |  | 1 |  | 2 | 1 |
| Middle Temporal Pole | L | 3 | 2 |  |  |  |  |
| Olfactory Gyrus | L | 4 |  | 1 |  |  |  |
| Olfactory Gyrus | R | 2 |  |  | 2 |  | 1 |
| Pallidum | L |  | 3 |  |  | 1 | 1 |
| Rolandic Operculum | R | 2 | 1 |  |  | 1 | 1 |
| Superior Frontal Gyrus | R | 1 |  | 2 |  | 1 | 1 |
| Supramarginal Gyrus | R | 1 | 2 | 1 |  |  | 1 |
| Amygdala | R | 1 |  |  |  |  | 3 |
| Inferior Frontal Gyrus, pars orbitalis | L | 1 |  | 1 |  | 1 | 1 |
| Inferior Frontal Gyrus, pars triangularis | R | 1 |  | 2 |  |  | 1 |
| Medial Frontal Gyrus | L |  | 2 |  |  |  | 2 |
| Middle Frontal Gyrus | R | 1 | 1 | 1 |  |  | 1 |
| Paracentral Lobule | R |  | 1 |  |  | 1 | 2 |
| ParaHippocampal Gyrus | R |  |  | 1 |  | 1 | 2 |
| Precentral Gyrus | L |  | 2 | 1 |  |  | 1 |
| Superior Frontal Gyrus | L | 1 |  | 1 | 1 |  | 1 |
| Superior Frontal Gyrus, orbital part | R | 1 |  |  | 1 | 1 | 1 |
| Superior Parietal Gyrus | R |  |  |  | 3 | 1 |  |
| Superior Temporal Gyrus | R |  |  |  |  | 2 | 2 |
| Supramarginal Gyrus | L |  | 1 | 1 |  | 1 | 1 |
| Inferior Frontal Gyrus, pars opercularis | R |  |  | 2 |  |  | 1 |
| Medial Orbitofrontal Cortex | L |  |  | 1 |  |  | 2 |
| Medial Orbitofrontal Cortex | R | 1 |  |  |  | 1 | 1 |
| Middle Frontal Gyrus | L | 1 | 1 |  |  | 1 |  |
| Middle Temporal Pole | R |  | 1 |  |  | 1 | 1 |
| ParaHippocampal Gyrus | L |  | 1 | 1 |  |  | 1 |
| Rectus Gyrus | R | 1 |  |  |  | 1 | 1 |
| Rolandic Operculum | L |  |  |  |  | 3 |  |
| Superior Frontal Gyrus, orbital part | L |  |  | 1 | 1 |  | 1 |
| Superior Parietal Gyrus | L | 1 |  |  | 1 |  | 1 |
| Superior Temporal Gyrus | L | 1 |  |  |  | 1 | 1 |
| Heschl Gyrus | L | 1 |  |  |  | 1 |  |
| Inferior Parietal Gyrus | R |  | 1 | 1 |  |  |  |
| Inferior Temporal Gyrus | L |  |  |  | 1 | 1 |  |
| Middle Temporal Gyrus | L |  | 1 |  |  |  | 1 |
| Posterior Cingulate Gyrus | L |  |  |  |  |  | 2 |
| Superior Temporal Pole | L | 1 | 1 |  |  |  |  |
| Supplementary Motor Area | L |  | 1 |  |  |  | 1 |
| Caudate Nucleus | L |  | 1 |  |  |  |  |
| Rectus Gyrus | L |  |  |  |  |  | 1 |
