# Supplementary figures and images for: Disconnection from others in autism is more than just a feeling: whole-brain neural synchrony in adults during implicit processing of emotional faces
Source: Mol Autism. 2017 Feb 22;8:7. doi: 10.1186/s13229-017-0123-2 (PMC5351200; doi:10.1186/s13229-017-0123-2)

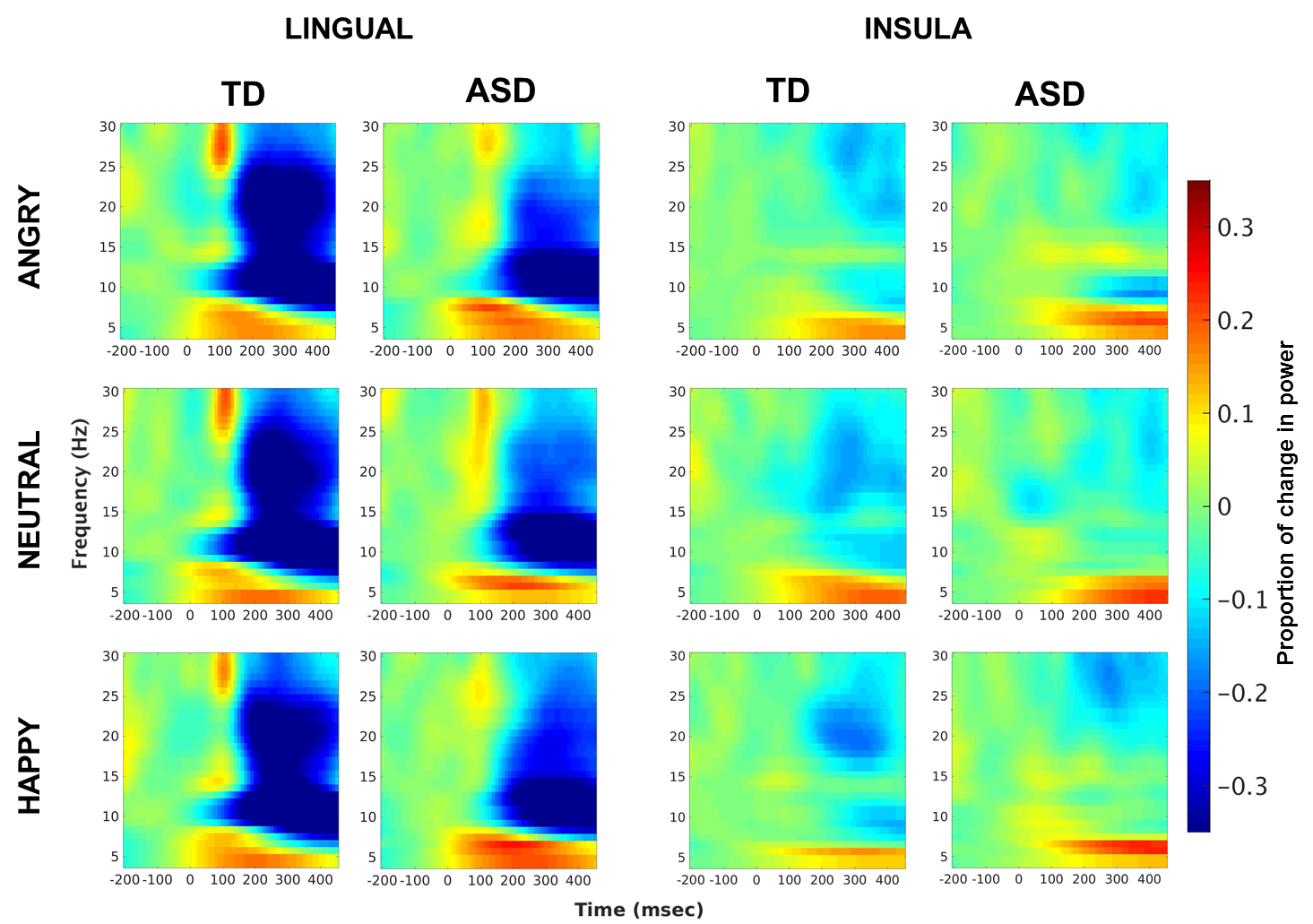

Supplement: Additional file 4: Figure S1. — Time-frequency activation: Beta de-synchronization in the left lingual gyrus and insula. (TIF 1151 kb) [file 13229_2017_123_MOESM4_ESM.tif]
